# Mitochondria Are Dynamically Transferring Between Human Neural Cells and Alexander Disease-Associated GFAP Mutations Impair the Astrocytic Transfer

**DOI:** 10.3389/fncel.2019.00316

**Published:** 2019-07-10

**Authors:** Longfei Gao, Zhen Zhang, Jing Lu, Gang Pei

**Affiliations:** ^1^State Key Laboratory of Cell Biology, CAS Center for Excellence in Molecular Cell Science, Shanghai Institute of Biochemistry and Cell Biology, Chinese Academy of Sciences, University of Chinese Academy of Sciences, Shanghai, China; ^2^Shanghai Key Laboratory of Signaling and Disease Research, Collaborative Innovation Center for Brain Science, School of Life Sciences and Technology, Tongji University, Shanghai, China; ^3^Institute for Stem Cell and Regeneration, Chinese Academy of Sciences, Beijing, China

**Keywords:** mitochondria, transfer, astrocytes, neuronal cells, GFAP mutation, Alexander disease

## Abstract

Mitochondria are the critical organelles for energy metabolism and cell survival in eukaryotic cells. Recent studies demonstrated that mitochondria can intercellularly transfer between mammalian cells. In neural cells, astrocytes transfer mitochondria into neurons in a CD38-dependent manner. Here, using co-culture system of neural cell lines, primary neural cells, and human pluripotent stem cell (hPSC)-derived neural cells, we further revealed that mitochondria dynamically transferred between astrocytes and also from neuronal cells into astrocytes, to which CD38/cyclic ADP-ribose signaling and mitochondrial Rho GTPases (MIRO1 and MIRO2) contributed. The transfer consequently elevated mitochondrial membrane potential in the recipient cells. By introducing Alexander disease (AxD)-associated hotspot mutations (R79C, R239C) into GFAP gene of hPSCs and subsequently inducing astrocyte differentiation, we found that GFAP mutations impaired mitochondrial transfer from astrocytes and reduced astrocytic CD38 expression. Thus, our study suggested that mitochondria dynamically transferred between neural cells and revealed that AxD-associated mutations in GFAP gene disrupted the astrocytic transfer, providing a potential pathogenic mechanism in AxD.

## Introduction

Mitochondria are critical organelles in eukaryotic cells. They are not only the major providers of cellular ATP and some macromolecules, but also important participants in calcium signaling, apoptosis, and other signaling pathways. Mitochondria were previously considered to be inherited from maternal lineage. However, this concept is challenged by increasing amounts of studies revealing that mitochondria can intercellularly transfer between mammalian cells, such as from stromal cells to damaged cells and from host cells to tumor cells (Islam et al., 2012; Tan et al., 2015). The functional consequences of the transfer range from promoting survival and recovery of damaged cells to enhancing metastasis and chemoresistance of tumor cells (Griessinger et al., 2017; Torralba et al., 2016). In the brain, one of the organs with high requirements of mitochondria-derived energy, mitochondria can transfer from astrocytes into neurons and contribute to the neuroprotection and neurorecovery after stroke (Hayakawa et al., 2016). Here, using *in vitro* co-culture system of astrocytes and neurons, the two most abundant brain cells, we further revealed intercellular mitochondrial transfer between astrocytes and from neuronal cells into astrocytes, suggesting that intercellular mitochondrial transfer might prevalently occur between neural cells.

Alexander disease (AxD) is a rare but fatal neurological disorder. It is mainly caused by the mutation of astrocyte specific intermediate filament GFAP (Olabarria and Goldman, 2017). However, how GFAP mutation leads to astrocyte disorder and AxD pathology has not been clearly elucidated. Recent study reveals that the distribution and function of endoplasmic reticulum and lysosome are disrupted in astrocytes with GFAP mutations (Jones et al., 2018). Several reports indicate that mitochondrial function may also be compromised in AxD (Caceres-Marzal et al., 2006; Nobuhara et al., 2004), though it has not been strictly examined with isogenic cell pairs. In this study, we introduced AxD-associated hotspot mutations into GFAP gene of human pluripotent stem cells (hPSCs) and subsequently induced astrocyte differentiation to generate astrocytes with GFAP mutations as previously reported (Canals et al., 2018). By comparing the mitochondrial transfer capacity between wildtype (WT) and GFAP-mutated astrocytes, we found that GFAP mutations impaired intercellular mitochondrial transfer from astrocytes, providing a perspective to dissect a potential pathogenic mechanism of the complicated neurological disorders.

## Materials and Methods

### Cell Culture

Human astrocyte cell line (HA) was purchased from ScienCell (#1800) and maintained in DMEM medium with 10% FBS. Human neuronal cell line SK-N-SH (SK) was purchased from ATCC and cultured in MEM medium with 10% FBS. H1 ESC was kindly provided by Dr. Duanqing Pei and cultured with mTeSR^TM^1 (StemCell, #85850) and Matrigel Matrix (Corning, #354277) following manufacturer’s instructions. The use of animals was approved by the Institutional Animal Care and Use Committee (IACUC) of Shanghai Institute of Biochemistry and Cell Biology, Chinese Academy of Sciences. C57BL/6 mouse of postnatal day 1 and both gender were used and purchased from Shanghai SLAC Laboratory Animal Co., Ltd. (Shanghai, China). Primary mouse astrocytes and neurons were isolated as previously described (Cheng et al., 2015). Astrocytes were maintained in DMEM/F12 medium supplemented with 2% B27 and 10% FBS and neurons were maintained in Neurobasal medium supplemented with 2% B27 and 1% glutamax. The medium was changed every 2 days. Brightfield images were captured with Zeiss Observer. Z1 microscope. Time lapse images were captured with Olympus FV10i microscope. Cells for time lapse live imaging were plated into Matrigel-pretreated Lab-Tek II Chambered Coverglass (Thermo Fisher Scientific, #155409PK). Images were captured at a 5 min interval after the cells were attached. The chamber of the microscope was maintained at 37°C and constantly bubbled with 5% CO_2_ / 20% O_2_ / 75% N_2_ mixture. For cADPR and CD38 inhibitors treatment, the chemicals were added 1 h after the co-cultured cells attached. The following final concentration were used: 2 mM cADPR (Sigma, #C7344), 30 μM quercetin (Selleck, #S2391), and 30 μM apigenin (Selleck, #2262). Cell cultures were routinely tested by PCR and confirmed to be free of mycoplasma contamination.

### Plasmids Construction and shRNA Sequences

The GFP-expressing lentiviral vector FuGW and NGN2 vector were used in previously studies (Gao et al., 2017). pLV-mitodsred vector was purchased from Addgene (#44386). SOX9 and NFIB vector were constructed by replacing the GFP with SOX9 cDNA (purchased from Sino Biological Inc.) and NFIB cDNA (kindly provided by Dr. Jiahuai Han) in FuGW vector. mitoBFP vector was constructed by (1) replacing dsred sequence with BFP sequence, which was derived from pCAG mito-mTagBFP2 plasmid (Addgene, #105011), in pLV-mitodsred plasmid; (2) replacing GFP with mitoBFP in FuGW. hGFAP::GFP vector was constructed by replacing the hUbC promoter in FuGW with human GFAP promoter (Cheng et al., 2015; Guo et al., 2014). hSYN1::mitoBFP vector was constructed by replacing the hUbC promoter in mitoBFP with human SYN1 promoter (kindly provided by Dr. Jiawei Zhou) (Zhu et al., 2015). CD38 overexpression vector was constructed by replacing GFP sequence in FuGW with CD38 cDNA (purchased from Kelei biological Technology, Shanghai). shRNA targeting CD38, MIRO1, or MIRO2 was inserted into pLKO.1 puro vector (Addgene, #8453) following the provider’s instructions. The shRNA sense sequences were as follows:

scramble, AGATCATGCGCTCTTAGTGCT;
shCD38-1, CCAAAGTGTATGGGATGCTTT;
shCD38-2, CCAGAGAAGGTTCAGACACTA;
shMIRO1-1, GTGGACGCTCACGACTTATTT;
shMIRO1-2, ATGATCCTTTGGGTTCTATAA;
shMIRO2-1, CGTCTACAAGCACCATTACAT;
shMIRO2-2, GCGTCTACAAGCACCATTACA.

### Lentivirus Preparation and Infection

Lentivirus was produced with HEK 293T cells as previously described (Gao et al., 2017). Briefly, the day before transfection, 8 million cells were seeded into a 10 cm dish in DMEM (with 10% FBS) medium. On the next day, lentiviral vectors was co-transfected into 293T cells with Vsv-g and Pax2 plasmids using calcium phosphate transfection. Medium was refreshed 6 h after transfection. The supernatant was collected and filtered with a 0.45 μm filter at 48 and 72 h after transfection. Lentivirus was concentrated with Lenti-concentin virus precipitation solution (Excellbio) following the manufacturer’s instructions. For the infection of H1 ESC, cells were dissociated into single cells with accutase and replated onto Matrigel pretreated plates with 2 μM Thiazovivin (MedChemExpress, #HY-13257). On the next day, concentrated lentivirus was diluted with fresh mTeSR medium and added to the cells. 4 μg/ml polybrene was also added to improve the infection efficiency. After incubated in the incubator for 1 h, the cells were washed with fresh DMEM/F12 medium for three times and maintained with fresh mTeSR medium. For infection of other cells in this study, concentrated lentivirus diluted in fresh medium containing 8 μg/ml polybrene was added. After incubated in the incubator for 6 h, the cells were washed with fresh DMEM/F12 medium for three times and maintained with fresh medium.

### Astrocyte and Neuron Differentiation From H1 ESCs

Astrocyte differentiation was induced as previously described (Canals et al., 2018). Briefly, H1 ESC were dissociated into single cells with Accutase (Gibco, #A1110501) and seeded into 6-well plates at a density of 1 × 10^5^ cells per well in mTeSR containing 2 μM Thiazovivin. The next day (day 0), cells were infected with lentivirus expressing NFIB and SOX9. On days 1 and 2, the medium was changed into expansion medium (DMEM/F12, 10% FBS, 1% N2 supplement, and 1% glutamax). Day 3, the medium was changed into 75% expansion medium and 25% FGF medium (Neurobasal, 2% B27 supplement, 1% non-essential amino acid, 1% glutamax, 10% FBS, 5 ng/ml CNTF, 10 ng/ml FGF, 10 ng/ml BMP4). Day 4, cells were maintained in 50% expansion medium and 50% FGF medium. Day 5, medium was changed into 75% expansion medium and 25% FGF medium. Day 6, cells were maintained in FGF medium. Cells were dissociated and replated on day 7 and maintained in FGF medium. From day 10, cells were maintained with maturation medium (DMEM/F12:Neurobasal = 1:1, 1% N2, 1% sodium pyruvate, 1% glutamax, 5 μg/ml N-acetyl-cysteine, 10 ng/ml CNTF, 10 ng/ml BMP4, 5 ng/ml heparin-binding EGF-like growth factor, 500 μg/ml dbcAMP) and cluture medium was half changed every 2 days. Astrocytes differentiated more than 1 week, when most cells were double positive for GFAP and S100B, were used for experiments.

Neuron differentiation was also performed as previously described (Zhang et al., 2013). Briefly, H1 ESC was seeded into 6-well plates at a density of 2 × 10^5^ cells per well in mTeSR containing 2 μM Thiazovivin. On the next (day 0), lentivirus expressing NGN2 was added to infect cells. On days 1 and 2, culture medium was changed into DMEM/F12 medium containing 1% N2, 1% non-essential amino acid, 10 ng/ml BDNF, 10 ng/ml GDNF, 10 ng/ml IGF, and 0.2 μg/ml laminin. On day 3, the medium was further changed into Neurobasal medium supplemented with 2% B27, 1% glutamax, 10 ng/ml BDNF, 10 ng/ml GDNF, 10 ng/ml IGF, and 0.2 μg/ml laminin. From day 4, the medium was half-changed every 2 days. Neurons differentiated for more than 7 days, when most cells exhibited a neuronal morphology and were positive for neuronal markers, were used for experiments.

### Immunofluorescence Staining

Immunostaining was performed as previously described (Gao et al., 2017). Briefly, cells for immunostaining were seeded onto Matrigel pretreated coverslips. After aspiration of medium, cells were fixed with 4% PFA for 15 min at room temperature. Then cells were treated with permeabilization and blocking buffer (1% BSA and 0.5% Triton-X 100 in PBS) for 1 h at room temperature. Primary antibodies were incubated overnight at 4°C. The next day, fluorescent-dye conjugated secondary antibodies were incubated for 1 h at room temperature. Slides were mounted and images were captured with Leica SP8 microscope. The following antibodies were used in this study: TOM20 (Santa Cruz, #sc-17764), ATP5A1 (Abclonal, #5884), LAMP1 (Abcam, #24170), OCT4 (Santa Cruz, #sc-9081), NANOG (Santa Cruz, #sc-33760), SOX2 (Santa Cruz, #sc-17320), S100B (Sigma, #S2532), GFAP (Dako, #Z033401; Thermo Fisher, #13-0300), CRYAB (Santa Cruz, #sc-137143), TUJ1 (Biolegend, #801202), DCX (Santa Cruz, #sc-8066), MAP2 (Millipore, #AB5622), NEUN (Millipore, #ABN78), and SYN1 (Millipore, #Ab1543).

### Flow Cytometry, Fluorescence Activated Cell Sorting, and TMRE Staining

Cells for flow cytometry analysis were dissociated into single cells by TrypLE (Thermo Fisher Scientific, #12604021) and resuspended with cold PBS buffer. BD LSRII or Beckman CytoFlex LX was used for flow cytometry analysis. Flowjo or CytExpert software were used for data analysis. Fluorescence activated cell sorting (FACS) was performed with BD Influx. For separation of GFP^+^dsred^+^ and GFP^+^dsred^–^ cells in Supplementary Figure S4, HA-GFP and HA-mitodsred cells were co-cultured for 48 h before FACS.

For TMRE staining, cells were washed with 0.2% FBS-containing DMEM/F12 for twice. Then cells were incubated with 500 nM TMRE (Thermo Fisher Scientific, #T669), which was diluted in 0.2% FBS-containing DMEM/F12, in the incubator for 30 min. After the incubation, cells were washed with 0.2% FBS-containing DMEM/F12 for twice and then dissociated into single cells with TrypLE. Beckman CytoFlex LX and CytExpert software were used to read and analysis TMRE intensity.

### Quantification of RNA and Mitochondrial DNA

RNA extraction and reverse transcription were performed with Trizol reagent (Sigma) and PrimeScript^TM^RT Master Mix (Takara, #RR036A) following manufacturer’s instructions. Quantitative real-time PCR was performed with HotStart SYBR Green qPCR Master Mix (Excell, #MB000-3013) and MX3000P Stratagene PCR machine. The gene expression levels were normalized to the internal control (HPRT). Total DNA was extracted from indicated cells with DNA extraction kit (Tiangen, #DP304). Quantitative PCR was performed as above-mentioned with specific primers. Mitochondrial copy number was calculated as the ratio of mitochondrial DNA to nuclear DNA. The following primers were used:

MIRO1-Forward, AAGGTAACAAGTCGATGGATTCC;
MIRO1-Reverse, TCAGGTTTTTCGCTGAACACT;
MIRO2-Forward, CCACAAGGCAAACGTGGTG;
MIRO2-Reverse, AGGTCTCAATCTCGGGAAACT;
CD38-Forward, AGACTGCCAAAGTGTATGGGA;
CD38-Reverse, GCAAGGTACGGTCTGAGTTCC;
COX1-Forward, CTTCGTCTGATCCGTCCTAATC;
COX1-Reverse, TTGAGGTTGCGGTCTGTTAG;
CYTB-Forward, AGACAGTCCCACCCTCACAC;
CYTB-Reverse, AAGAGAAGTAAGCCGAGGGC;
PGC1a-Forward, CCTGTGGATGAAGACGGATT;
PGC1a-Reverse, TAGCTGAGTGTTGGCTGGTG;
PPARa-Forward, GCTTTGGCTTTACGGAATA;
PPARa-Reverse, TCCCGACAGAAAGGCACT;
TFAM-Forward, GATGCTTATAGGGCGGAG;
TFAM-Reverse, GCTGAACGAGGTCTTTTTGG;
NRF1-Forward, GATCGTCTTGTCTGGGGAAA;
NRF1-Reverse, GGTGACTGCGCTGTCTGATA;
NRF2a-Forward, TAGACCTCACCACACTCAAC;
NRF2a-Reverse, GTGACCAAACGGTTCAACTC;
HPRT-Forward, CCTGGCGTCGTGATTAGTGAT;
HPRT-Reverse, AGACGTTCAGTCCTGTCCATAA;
Human mtDNA-F, CCTTCTTACGAGCCAAAA;
Human mtDNA-R, CTGGTTGAACATTGTTTG (Sokolova et al., 2004);
Human nDNA-F, GAGTGGGAATCAGAGCTTCACGGGT;
Human nDNA-R, CCACGTCATTTACAGCATTTCAATG (Hobbs et al., 1992).

### Western Blot

Western blot was performed as previously described (Hu et al., 2015). Cells were washed with cold PBS and lysed with RIPA lysis buffer (Beyotime). Protein concentration was measured with Pierce^TM^ BCA Protein Assay Kit (Thermo Fisher Scientific, #23227) and adjusted with Laemmli’s sample buffer. Proteins were denatured at 95°C for 5 min. Cell samples were separated on SDS-PAGE and transferred onto nitrocellulose membrane. Primary antibodies include rabbit anti-MIRO1 (1: 500, Abclonal, #A5838), rabbit anti-MIRO2 (1:300, Cell Signaling Technology, #14016), rabbit anti-CD38 (1:500, Abclonal, #A13611), and rabbit anti-actin (1:1000, sigma, #A2066). HRP-conjugated secondary antibody and Clarity^TM^ Western ECL Substrate were used to detect the membrane with MiniChemi^TM^ Chemiluminescence imager (Sagecreation).

### CRISPR/Cas9 Gene Editing

H1 ESCs were dissociated into single cells with accutase and re-plated onto Matrigel pretreated 6-well plate with 2 μM Thiazovivin. On the next day, 1 μg px330-mcherry (Addgene, #98750, kindly provided by Dr. Jinsong Li) inserted with sgRNA and 2.5 μg donor vector (T vectors with mutations and recombination homologous arms) were transfected into each well with 8 μl FuGene (Promega, #2311) following the manufacturer’s instructions. 24 h after transfection, mcherry-positive H1 cells were purified by FSCS (BD Influx) and plated onto Matrigel pretreated 10-cm plate with 2 μM Thiazovivin. Single colony was picked about 10 days after sorting. The mutations were validated with PCR and sequencing. The following oligonucleotides were used:

R79C-sgRNA1, TCTCGATGTAGCTGGCAAAG;
R79C-sgRNA2, CATCGAGAAGGTTCGCTTCC;
R79C-sgRNA3, GCGAACCTTCTCGATGTAGC;
R79C-Forward, CTCAGCCCTTTCCTTCCTTT;
R79C-Reverse, CGCTTCCAACTCCTCCTTTAT;
R79C_Mutation-Forward, ATGACTGCTTTGCCAGC;
R79C_Mutation-Reverse, GCTGGCAAAGCAGTCAT;
R239C-sgRNA1, ACTGCGTGCGGATCTCTTTC (Canals et al., 2018);
R239C-sgRNA2, ACATGCATGAAGCCGAAGAG (Canals et al., 2018);
R239C-sgRNA3, CTGCGTGCGGATCTCTTTCA;
R239C-Forward, CTTAATCCTCCTGCTGCTCTAC;
R239C-Reverse, GTTCTCTACGGGCACTATGTT;
R239C_Mutation-Forward, AGATCTGCACGCAGTATG;
R239C_Mutation-Reverse, CATACTGCGTGCAGATCT.

### Statistical Analysis

All quantified data were presented as mean ± SEM and statistically analyzed. Two-tailed student *t* test was used to calculate the statistical significance with *p* values between two groups. One-way ANOVA followed Dunnett’s multiple comparison test was applied to calculate the statistical significance with *p* values among three or more groups. The statistical values and used methods were specified in the figure legends. A *p* value less than 0.05 was considered as significant different. ^*^, *p* < 0.05; ^∗∗^, *p* < 0.01; ^∗∗∗^, *p* < 0.001.

## Results

### Intercellular Mitochondrial Transfer Between Astrocytes

**FIGURE 1:**
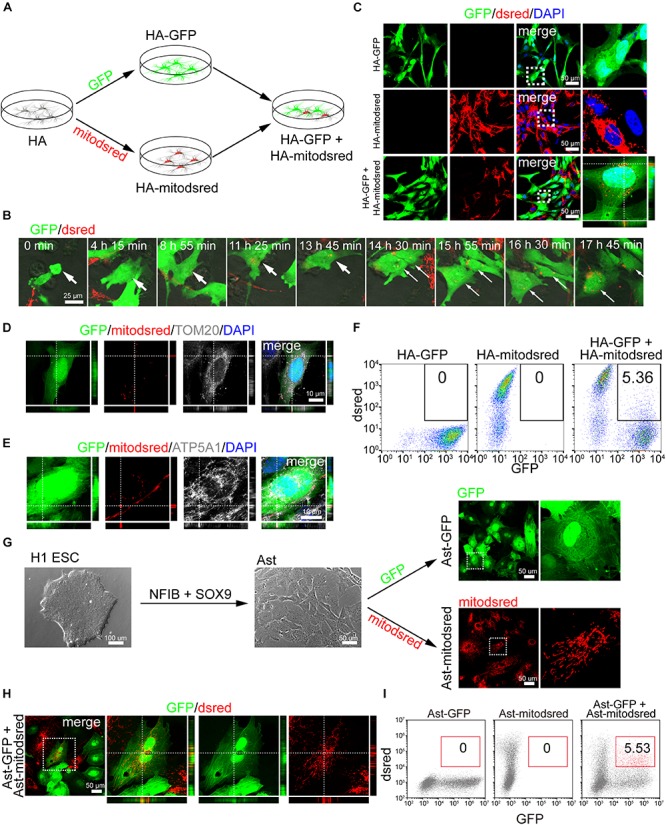
Intercellular mitochondrial transfer between astrocytes. **(A)** Schematic showing that human astrocytes (HA) were labeled with GFP or mitodsred and then co-cultured at a ratio of 1:1. **(B)** Time-lapse live images showing mitochondria from HA-mitodsred dynamically transferring into HA-GFP. **(C)** Confocal images showing dsred-labeled mitochondria in HA-GFP after co-cultured for 24 h. **(D,E)** Immunostaining of TOM20 **(D)** and ATP5A1 **(E)** on transferred mitochondria in HA after co-cultured for 24 h. **(F)** Flow cytometry analyzing mitochondrial transfer efficiency between HA after co-cultured for 24 h. **(G)** Astrocytes (Ast) were derived from H1 ESCs with NFIB and SOX9 and then labeled with GFP or mitodsred lentivirus. **(H)** Confocal images showing that mitochondria from Ast-mitodsred were observed in Ast-GFP after co-cultured for 24 h. **(I)** Flow cytometry analyzing mitochondrial transfer efficiency between Ast after co-cultured for 24 h.

To label mitochondria, we infected human astrocyte (HA) cell line with lentivirus expressing mitochondria-localized dsred (mitodsred) (Figure 1A), which specifically labeled mitochondria as shown by co-staining of MitoTracker and TOM20, a mitochondrial marker (Supplementary Figures S1A,B). Then the mitodsred-labeled HA (HA-mitodsred) were co-cultured with another group of HA, which were labeled with GFP, at a ratio of 1:1 (Figure 1A). Within 24 h, mitochondria from HA-mitodsred were dynamically transferred into GFP-labeled HA (HA-GFP) as showed by time lapse live imaging and confocal images (Figures 1B,C). The transferred mitochondria were inside of HA-GFP, but not merely associated with the surface of HA-GFP, as showed by confocal z-stack images (Figure 1C). Moreover, transferred mitochondria were positive for TOM20 and ATP5A1, another mitochondrial marker (Figures 1D,E). The mitochondrial transfer efficiency (percentage of GFP^+^dsred^+^ cells in the co-culture system) was about 5% after co-cultured for 24 h (Figure 1F). These data suggested that intercellular mitochondrial transfer occurred between HA.

To further explore whether mitochondria transferred between other types of astrocytes, we differentiated H1 ESCs, which were confirmed to be positive for pluripotency markers OCT4, NANOG, and SOX2 by immunostaining (Supplementary Figures S1C–E), into astrocytes with NFIB and SOX9 as previously described (Canals et al., 2018). The percentage of GFAP^+^S100B^+^ cells in differentiated astrocytes (Ast) were about 80% (Supplementary Figures S1F,G, 5C). GFP or mitodsred virus was used to label Ast as mentioned above (Figure 1G) and then the differentially labeled cells were co-cultured at a ratio of 1:1. Mitochondria from Ast-mitodsred were detected in Ast-GFP and the transfer efficiency was about 5% after co-cultured for 24 h (Figures 1H,I).

We also isolated primary astrocytes from the cortex of postnatal mouse (Supplementary Figure S1H) and confirmed that nearly all the cells were positive for astrocyte marker S100B (Supplementary Figure S1I). Then we labeled mouse astrocytes (mAst) with mitodsred or GFP and co-cultured the distinctly labeled mAst as above-mentioned (Supplementary Figure S1H). Mitochondria from mAst-mitodsred were detected in mAst-GFP after co-cultured for 24 h (Supplementary Figures S1J,K). When HAs and mouse astrocytes were co-cultured at 1:1, mitochondrial transfer from mouse astrocytes into HAs (Supplementary Figure S1L) and from HAs into mouse astrocytes (Supplementary Figure S1M) were also observed after co-cultured for 24 h. Collectively, these data demonstrated that mitochondria were dynamically transferring between astrocytes.

### Mitochondrial Transfer From Neuronal Cells Into Astrocytes

**FIGURE 2:**
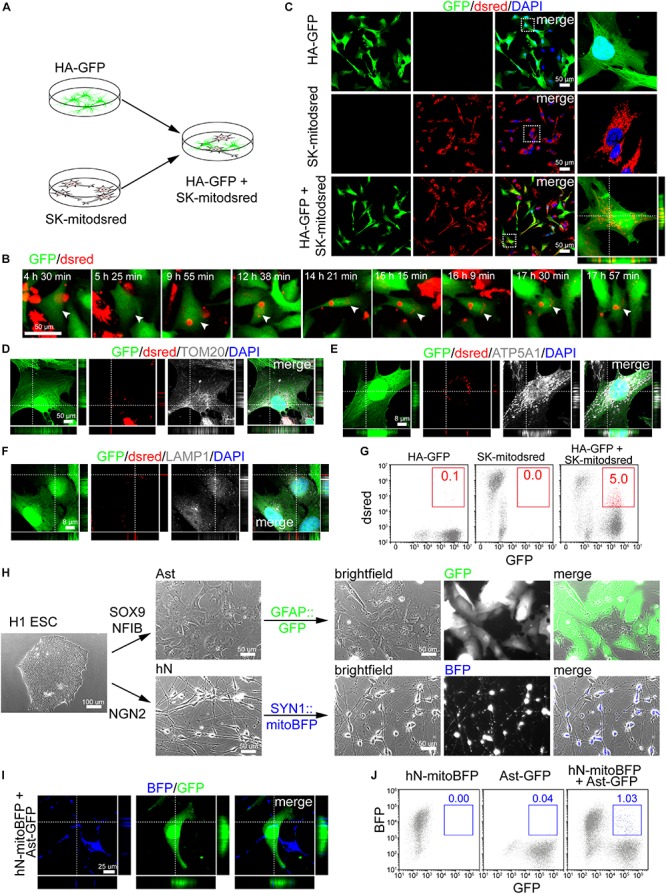
Mitochondrial transfer from neuronal cells into astrocytes. **(A)** Schematic showing that HA-GFP were co-cultured with SK-mitodsred at a ratio of 1:1. **(B)** Time-lapse live images showing mitochondria from SK-mitodsred dynamically transferring into HA-GFP. **(C)** Confocal images showing mitochondria from SK-mitodsred in HA-GFP after co-cultured for 24 h. **(D–F)** Immunostaining of TOM20 **(D)**, ATP5A1 **(E)**, and LAMP1 **(F)** after co-cultured for 24 h. **(G)** Flow cytometry analyzing mitochondrial transfer efficiency from SK into HA after co-cultured for 24 h. **(H)** H1 ESC-derived astrocytes (Ast) or neurons (hN) were labeled with GFAP::GFP or SYN1::mitoBFP. **(I)** Confocal images showing that mitochondria from hN-mitoBFP were observed in Ast-GFP after co-cultured for 48 h. **(J)** Flow cytometry analyzing mitochondrial transfer efficiency from hN cells into Ast after co-cultured for 48 h.

HA-GFP were co-cultured with mitodsred-labeled human neuronal cell line SK (SK-mitodsred) at a ratio of 1:1 (Figure 2A). Within 24 h of co-culture, dsred-labeled mitochondria were transferring into HA-GFP (Figure 2B) and confirmed to be inside of HA-GFP by confocal z-stack images (Figure 2C). Transferred mitochondria were positive for TOM20 and ATP5A1 (Figures 2D,E), but negative for lysosomal marker LAMP1 (Figure 2F), which suggested that unlike previous report (Davis et al., 2014), the transferred mitochondria here might not be degraded. Flow cytometry showed that about 5% cells were double positive for GFP and dsred in the co-culture system (Figure 2G).

Besides aforementioned Ast, we also generated neuronal cells from H1 ESCs by NGN2 (Zhang et al., 2013; Figure 2H). Immunostaining performed on day 7 cells showed that differentiated neurons (hN) were positive for TUJ1, MAP2, NEUN, and SYN1 (Supplementary Figures S2A–D). hSYN1::mitoBFP lentivirus was used to label mitochondria in hN and hGFAP::GFP to label Ast (Figure 2H). Immunostaining showed that BFP-labeled neurons (hN-mitoBFP) were positive for TUJ1, MAP2, and NEUN (Supplementary Figures S2E,F) and GFP-labeled astrocytes (Ast-GFP) were positive for GFAP and S100B (Supplementary Figure S2G). Then Ast-GFP were dissociated and re-plated onto hN-mitoBFP at a ratio of approximately 1:1. After co-cultured for 48 h, BFP^+^ mitochondria were observed inside of the Ast-GFP (Figure 2I). The transfer efficiency was about 1%, lower than that on cell lines (Figure 2J).

We also isolated primary neurons (mNeuron) from the cortex of postnatal mouse (Supplementary Figure S2H). Judging from cell morphology and TUJ1 staining, the purity of mNeuron was above 90% (Supplementary Figure S2I). Then we used GFP or mitodsred to label above-mentioned mAst or mitochondria in mNeuron, respectively (Supplementary Figure S2H). When dissociated mAst-GFP were re-plated onto mNeuron-mitodsred, mitochondrial transfer from mNeuron into mAst was detected after 48 h of co-culture (Supplementary Figure S2J). Taken together, our data suggested that neuronal mitochondria were also dynamically transferring into astrocytes.

### Contribution of CD38/Cyclic ADP-Ribose (cADPR) Signaling and Mitochondrial Rho GTPases to the Transfer

**FIGURE 3:**
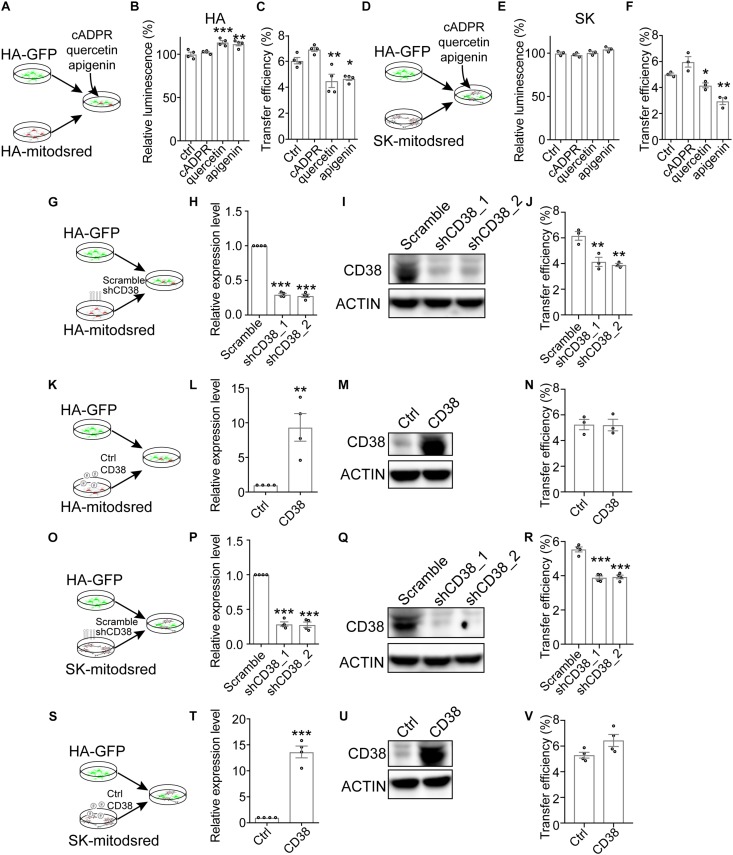
Contribution of CD38/cADPR signaling to the transfer **(A–F)**. Effects of cADPR or CD38 inhibitors (quercetin, apigenin) on mitochondrial transfer between neural cells. **(A)** Schematic showing that co-cultured HA-GFP and HA-mitodsred cells were treated with cADPR, quercetin, or apigenin. **(B)** Analysis of cell viability on HA with indicated treatment for 24 h by CellTiter Glo. (Adjusted *p* values: ctrl vs. cADPR, 0.7403; ctrl vs. quercetin, 0.0009; and ctrl vs. apigenin, 0.0029). **(C)** Flow cytometry analyzing mitochondrial transfer efficiency between HA with indicated chemical treatment for 24 h. (Adjusted *p* values: ctrl vs. cADPR, 0.1478; ctrl vs. quercetin, 0.0091; and ctrl vs. apigenin, 0.0194). **(D)** Schematic showing that co-cultured HA-GFP and SK-mitodsred cells were treated with cADPR, quercetin, or apigenin. **(E)** Analysis of cell viability on SK with indicated treatment for 24 h by CellTiter Glo. (Adjusted *p* values: ctrl vs. cADPR, 0.7573; ctrl vs. quercetin, 0.9783; and ctrl vs. apigenin, 0.1579). **(F)** Flow cytometry analyzing mitochondrial transfer efficiency from SK into HA with indicated chemical treatment for 24 h. (Adjusted *p* values: ctrl vs. cADPR, 0.0775; ctrl vs. quercetin, 0.01263; and ctrl vs. apigenin, 0.0015). **(G–N)** Effects of CD38 in HA-mitodsred on mitochondrial transfer between HA. **(G)** Schematic showing that HA-GFP were co-cultured with indicated HA-mitodsred. **(H,I)**. RT-qPCR **(H)** and western blot **(I)** analysis of CD38 expression on HA-mitodsred with indicated shRNA transduction. (Adjusted *p* values in H: Scramble vs. shCD38_1, <0.001; Scramble vs. shCD38_2, <0.001). **(J)** Flow cytometry analyzing mitochondrial transfer efficiency from indicated HA-mitodsred into HA-GFP after co-cultured for 24 h. (Adjusted *p* values: Scramble vs. shCD38_1, 0.0048; Scramble vs. shCD38_2, 0.0027). **(K)** Schematic showing that HA-GFP were co-cultured with indicated HA-mitodsred. **(L,M)** RT-qPCR **(L)** and western blot **(M)** analysis of CD38 expression on HA-mitodsred with indicated lentiviral infection. (*p* = 0.0059 in **M**). **(N)** Flow cytometry analyzing mitochondrial transfer efficiency from indicated HA-mitodsred into HA-GFP after co-cultured for 24 h. (*p* = 0.9483). **(O–V)** Effects of CD38 in SK-mitodsred on mitochondrial transfer from SK into HA. **(O)** Schematic showing that HA-GFP were co-cultured with indicated SK-mitodsred. **(P,Q)** RT-qPCR **(P)** and western blot **(Q)** analysis of CD38 expression on SK-mitodsred with indicated shRNA transduction. (Adjusted *p* values in P: Scramble vs. shCD38_1, <0.001; Scramble vs. shCD38_2, <0.001). **(R)** Flow cytometry analyzing mitochondrial transfer efficiency from indicated SK-mitodsred into HA-GFP after co-cultured for 24 h. (Adjusted *p* values: Scramble vs. shCD38_1, *p* < 0.001; Scramble vs. shCD38_2, *p* < 0.001). **(S)** Schematic showing that HA-GFP were co-cultured with indicated SK-mitodsred. **(T,U)** RT-qPCR **(T)** and western blot **(U)** analysis of CD38 expression on SK-mitodsred with indicated lentiviral infection. (*p* < 0.001) **(V)** Flow cytometry analyzing mitochondrial transfer efficiency from indicated SK-mitodsred into HA-GFP after co-cultured for 24 h. (*p* = 0.0697) Data are represented as mean ± SEM. One-way ANOVA followed Dunnett’s multiple comparisons test was applied in **B,** **C,** **E,** **F,** **H,** **J,** **P,** **R**; Two-tailed student *t* test was used in **L,** **N,** **T,** **V**. ^*^p < 0.05, ^∗∗^*p* < 0.01, and ^∗∗∗^*p* < 0.001.

Previous study demonstrates that transfer of mitochondria from astrocytes into neurons and from bone marrow stromal cells into multiple myeloma cells is mediated by CD38, a transmembrane glycoprotein catalyzing the production of cADPR (Hayakawa et al., 2016; Marlein et al., 2019). To explore whether CD38/cADPR signaling involved here, we treated co-cultured HA-GFP and HA-mitodsred with cADPR or CD38 inhibitor (quercetin or apigenin) (Escande et al., 2013; Kellenberger et al., 2011; Figure 3A). The treatment of cADPR, quercetin, or apigenin for 24 h did not reduce cell viability as indicated by cellular ATP levels (Figure 3B). However, mitochondrial transfer efficiency was significantly inhibited by quercetin or apigenin (Figure 3C and Supplementary Figure S3A). Similarly, we also found that quercetin and apigenin significantly inhibited mitochondrial transfer from SK into HA without affecting cell viability (Figures 3D–F and Supplementary Figure S3B). The addition of cADPR slightly, but not significantly, promoted the transfer efficiency (Figures 3C,F). We also constructed CD38-targeting shRNA to genetically inhibit the expression of CD38 in HA-mitodsred cells (Figures 3G–I and Supplementary Figure S6), and found that consistent with the effect of pharmacological inhibition, genetic inhibition of CD38 also reduced the transfer efficiency (Figure 3J and Supplementary Figure S3C). However, overexpression of CD38 did not promote the transfer (Figures 3K–N and Supplementary Figure S3D). Similar results were observed when the expression level of CD38 was genetically inhibited or increased in SK-mitodsred and then the SK-mitodsred were co-cultured with HA-GFP (Figures 3O–V and Supplementary Figures S3E,F). These data collectively indicated that CD38/cADPR might be necessary, but not sufficient, for the mitochondrial transfer between neural cells.

**FIGURE 4:**
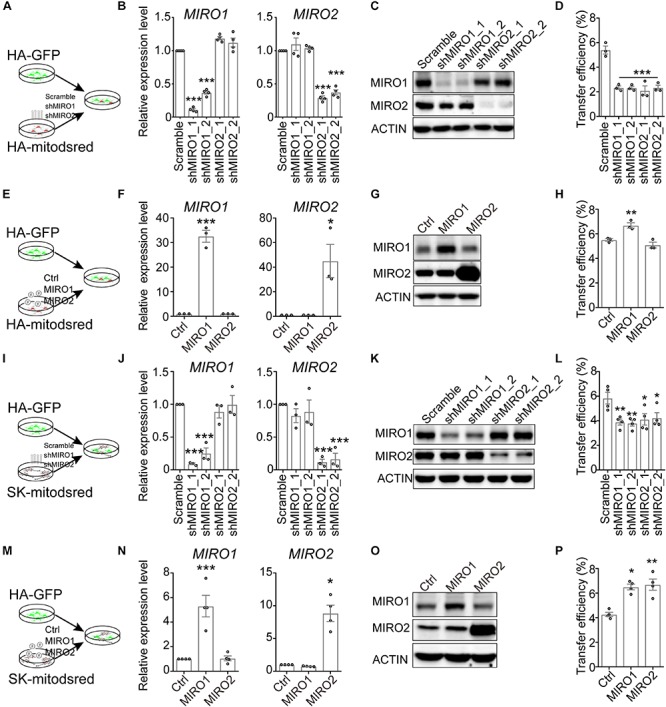
Contribution of mitochondrial Rho GTPases to the transfer. **(A–H)** Effects of MIRO1 and MIRO2 in HA-mitodsred on mitochondrial transfer between HA. **(A)** Schematic showing that HA-GFP were co-cultured with indicated HA-mitodsred. **(B,C)** RT-qPCR **(B)** and western blot **(C)** analysis of MIRO1 and MIRO2 expression on HA-mitodsred with indicated shRNA transduction. (Adjusted *p* values in B: *MIRO1*, Scramble vs. shMIRO1_1, <0.001; Scramble vs. shMIRO1_2, <0.001; Scramble vs. shMIRO2_1, 0.056; Scramble vs. shMIRO2_2, 0.0721. *MIRO2*, Scramble vs. shMIRO1_1, 0.4154; Scramble vs. shMIRO1_2, 0.9288; Scramble vs. shMIRO2_1, <0.001; Scramble vs. shMIRO2_2, <0.001). **(D)** Flow cytometry analyzing mitochondrial transfer efficiency from indicated HA-mitodsred into HA-GFP after co-cultured for 24 h. (Adjusted *p* values vs. Scramble <0.001) **(E)** Schematic showing that HA-GFP were co-cultured with indicated HA-mitodsred. **(F,G)** RT-qPCR **(F)** and western blot **(G)** analysis of MIRO1 and MIRO2 expression on HA-mitodsred with indicated lentiviral infection. (Adjusted *p* values in F: *MIRO1*, Ctrl vs. MIRO1, <0.001; ctrl vs. MIRO2, >0.999; *MIRO2* Ctrl vs. MIRO1, >0.999; ctrl vs. MIRO2, 0.0127). **(H)** Flow cytometry analyzing mitochondrial transfer efficiency from indicated HA-mitodsred into HA-GFP after co-cultured for 24 h. (Adjusted *p* values: Ctrl vs. MIRO1, 0.009; ctrl vs. MIRO2, 0.2953). **(I–P)** Effects of MIRO1 or MIRO2 in SK-mitodsred on mitochondrial transfer from SK into HA. **(I)** Schematic showing that HA-GFP were co-cultured with indicated SK-mitodsred. **(J,K)** RT-qPCR **(J)** and western blot **(K)** analysis of MIRO1 and MIRO2 expression on SK-mitodsred with indicated shRNA transduction. (Adjusted *p* values in J: *MIRO1*, Scramble vs. shMIRO1_1, <0.001; Scramble vs. shMIRO1_2, <0.001; Scramble vs. shMIRO2_1, 0.7474; Scramble vs. shMIRO2_2, >0.999. *MIRO2*, Scramble vs. shMIRO1_1, 0.5991; Scramble vs. shMIRO1_2, 0.8561; Scramble vs. shMIRO2_1, <0.001; Scramble vs. shMIRO2_2, <0.001). **(L)** Flow cytometry analyzing mitochondrial transfer efficiency from indicated SK-mitodsred into HA-GFP after co-cultured for 24 h. (Adjusted *p* values: Scramble vs. shMIRO1_1, 0.0084; Scramble vs. shMIRO1_2, 0.006; Scramble vs. shMIRO2_1, 0.0189; Scramble vs. shMIRO2_2, 0.0302). **(M)** Schematic showing that HA-GFP were co-cultured with indicated SK-mitodsred. **(N,O)** RT-qPCR **(N)** and western blot **(O)** analysis of MIRO1 and MIRO2 expression on SK-mitodsred with indicated lentiviral infection. (Adjusted *p* values in N: *MIRO1*, Ctrl vs. MIRO1, <0.001; ctrl vs. MIRO2, 0.9969; *MIRO2* Ctrl vs. MIRO1, 0.9599; ctrl vs. MIRO2, 0.0127). **(P)** Flow cytometry analyzing mitochondrial transfer efficiency from indicated SK-mitodsred into HA-GFP after co-cultured for 24 h. (Adjusted *p* values: Ctrl vs. MIRO1, 0.0119; ctrl vs. MIRO2, 0.005). Data are represented as mean ± SEM. One-way ANOVA followed Dunnett’s multiple comparisons test was applied in **B**, **D**, **F**, **H**, **J**, **L**, **N**, **P**. ^*^p < 0.05, ^∗∗^*p* < 0.01, and ^∗∗∗^*p* < 0.001.

Aside from CD38, MIRO1, one of the two mitochondrial Rho GTPases, also regulates mitochondrial transfer from mesenchymal stem cells into epithelial cells and cardiomyocytes (Ahmad et al., 2014; Zhang et al., 2016). To assess whether mitochondrial Rho GTPase affected the mitochondrial transfer here, we constructed shRNAs targeting MIRO1 or MIRO2, another mitochondrial Rho GTPase. Compared to scramble shRNA, MIRO1- or MIRO2-targeting shRNA reduced the expression level of MIRO1 or MIRO2 in HA (Figures 4A–C and Supplementary Figure S6). When HA-mitodsred were infected with MIRO1- or MIRO2-targeting shRNAs and then co-cultured with HA-GFP for 24 h (Figure 4A), mitochondrial transfer efficiency was reduced by MIRO1- or MIRO2-targeting shRNAs (Figure 4D and Supplementary Figure S3G). On the contrast, when MIRO1 was overexpressed in HA-mitodsred, mitochondrial transfer efficiency from HA-mitodsred into HA-GFP increased (Figures 4E–H and Supplementary Figure S3H). However, when MIRO1 or MIRO2 was down-regulated or up-regulated in HA-GFP, the transfer efficiency from HA-mitodsred into HA-GFP was not altered (Supplementary Figures S3I–L). We also assessed the effects of MIRO1 and MIRO2 on transfer of neuronal mitochondria into astrocytes. When shRNAs targeting MIRO1 or MIRO2 were transduced on SK-mitodsred, the expression level of MIRO1 or MIRO2 in SK-mitodsred was also reduced (Figures 4J,K). These SK-mitodsred were then co-cultured with HA-GFP (Figure 4I) and the transfer efficiency from SK into HA was reduced by MIRO1 or MIRO2 knockdown (Figure 4L and Supplementary Figure S3M). On the other hand, the efficiency was increased when MIRO1 or MIRO2 was overexpressed in SK-mitodsred (Figures 4M–P and Supplementary Figure S3N). Taken together, these data suggested that CD38/cADPR signaling and the two mitochondria-localized Rho GTPases contributed to the intercellular mitochondrial transfer between neural cells.

### Elevation of Mitochondrial Membrane Potential (MMP) in Cells With Transferred Mitochondria

**FIGURE 5:**
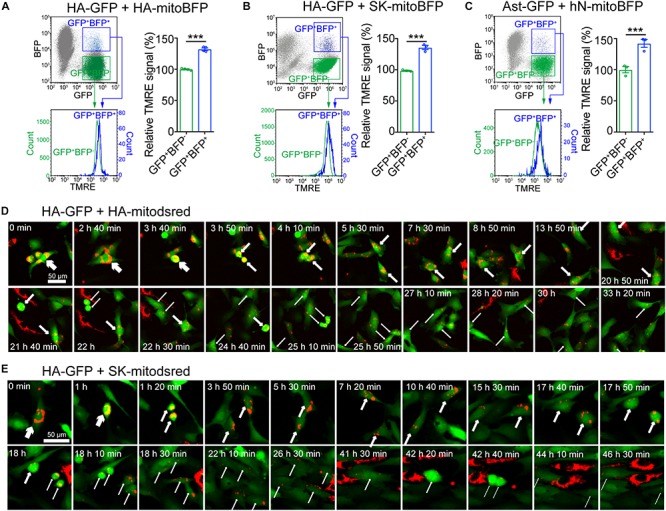
Elevation of mitochondrial membrane potential (MMP) in cells with transferred mitochondria **(A–C)** Measurement of MMP on co-cultured HA-GFP and HA-mitoBFP **(A)**, HA-GFP and SK-mitoBFP **(B)**, and Ast-GFP and hN-mitoBFP **(C)** by TMRE staining. TMRE signal intensity was compared between gated GFP^+^BFP^+^ and GFP^+^BFP^–^ cells. **(D,E)** Time-lapse live images showing the dynamics of mitochondria transferred from HA-mitodsred **(D)** or from SK-mitodsred **(E)** into HA-GFP along cell division. Data are represented as mean ± SEM. *P* values in **A,** **B,** **C** calculated with two-tailed student *t* test were <0.001. ^∗∗∗^*p* < 0.001.

To assess the effects of mitochondrial transfer on recipient cells, we measured MMP by tetramethylrhodamine ethyl ester perchlorate (TMRE) staining (Crowley et al., 2016). HA transduced with mitoBFP (HA-mitoBFP) were co-cultured with HA-GFP for 24 h and then TMRE staining was performed on co-cultured cells. The results showed that HA-GFP with transferred mitochondria (GFP^+^BFP^+^) had a relative higher TRME signal than HA-GFP without transferred mitochondria (GFP^+^BFP^–^) (Figure 5A). Similarly, mitochondrial transfer from SK or hNeuron also elevated the TMRE signal of recipient HA-GFP or Ast-GFP, respectively (Figures 5B,C). HA-GFP with transferred mitochondria (GFP^+^dsred^+^) and HA-GFP without transferred mitochondria (GFP^+^dsred^–^) were further separated from HA-GFP and HA-mitodsred co-cultured cells by FACS (Supplementary Figure S4B) and the expression levels of mitochondrial biogenesis and metabolism markers in GFP^+^dsred^+^ and GFP^+^dsred^–^ cells were analyzed. The results showed that except that *NRF1* was significantly, though slightly, upregulated in GFP^+^dsred^+^, there were no significant differences in the expression levels of other markers between GFP^+^dsred^+^ and GFP^+^dsred^–^ cells (Supplementary Figure S4C). These data collectively demonstrated that mitochondrial transfer might not impair, but instead slightly enhance, mitochondrial function of the recipient cells.

To assess the effects of mitochondrial transfer on cell cycle of the recipient cells, we performed time lapse live imaging on co-cultured HA-GFP and HA-mitodsred. These cells were co-cultured for 48 h to accumulate enough mitochondrial transfer. Then they were dissociated and re-plated and live imaging was performed once the cells attached. During the imaging periods, HA-GFP went through cell division and the transferred mitochondria distributed into the daughter cells during the division (Figure 5D). There was no significant difference in cell cycle duration between GFP^+^dsred^+^ and GFP^+^dsred^–^ cells (Supplementary Figure S4A). Similarly, HA-GFP with mitochondria transferred from SK-mitodsred also went through cell division normally and the transferred mitochondria also distributed into the daughter cells during the division (Figure 5E). These data indicated that mitochondrial transfer might not affect normal cell cycle progression of the recipient cells.

### Impaired Mitochondrial Transfer From Astrocytes With AxD-Associated GFAP Mutations

**FIGURE 6:**
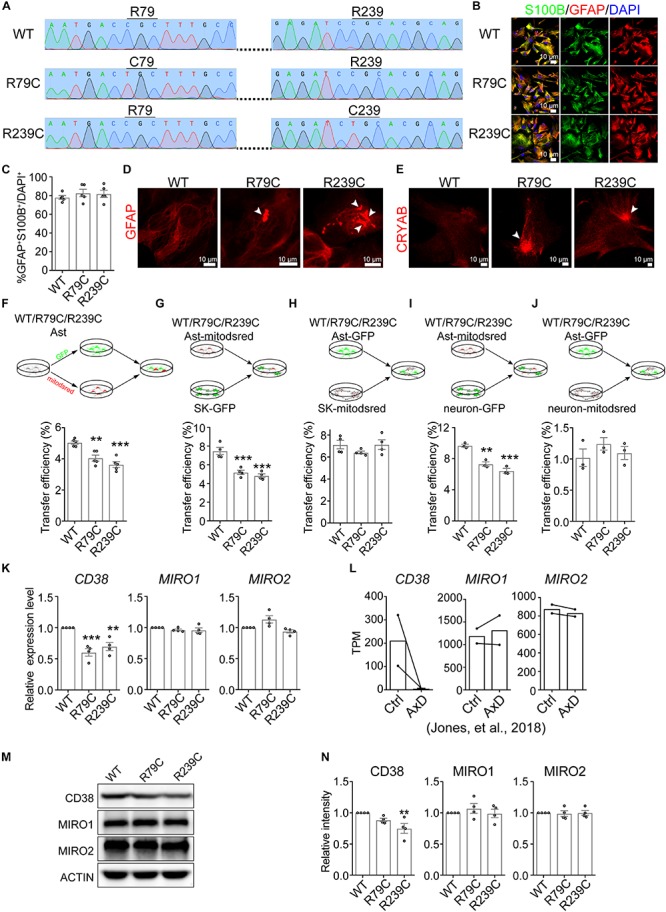
Impaired mitochondrial transfer from astrocytes with GFAP mutation. **(A)** Sanger sequencing validating R79C or R239C mutation in GFAP gene in H1 ESCs. **(B)** Immunostaining of S100B and GFAP on astrocytes derived from WT or GFAP-mutated ESCs. **(C)** Quantification of the percentage of GFAP^+^S100B^+^ cells in astrocytes derived from WT or GFAP-mutated ESCs. (Adjusted *p* values: WT vs. R79C, 0.5332; WT vs. R239C, 0.6487). **(D,E)** Representative images showing that GFAP aggregates **(D)** and CRYAB **(E)** (indicated by white arrowheads) were observed in R79C or R239C astrocytes, but rarely detected in WT astrocytes. **(F–J)** Flow cytometry analyzing mitochondrial transfer efficiency between indicated hESC-derived astrocytes **(F)**, from indicated hESC-derived astrocytes into SK cells **(G)**, from SK cells into indicated hESC-derived astrocytes **(H)**, from indicated hESC-derived astrocytes into neurons **(I)**, and from neurons into indicated hESC-derived astrocytes **(J)**. Cells co-cultured for 24 h were analyzed. (Adjusted *p* values in F: WT vs. R79C, 0.002; WT vs. R239C, <0.001. in G: WT vs. R79C, <0.001; WT vs. R239C, <0.001. in H: WT vs. R79C, 0.2924; WT vs. R239C, 0.9998. in I: WT vs. R79C, 0.0013; WT vs. R239C, <0.001. in J: WT vs. R79C, 0.3566; WT vs. R239C, 0.8587). **(K)** RT-qPCR analyzing the expression levels of *CD38*, *MIRO1*, and *MIRO2* on WT, R79C, or R239C astrocytes. (Adjusted *p* values: *CD38*, WT vs. R79C, <0.001; WT vs. R239C, 0.0041. *MIRO1*, WT vs. R79C, 0.3915; WT vs. R239C, 0.3568. *MIRO2*, WT vs. R79C, 0.0565; WT vs. R239C,0.4048). **(L)** TPM values of *CD38*, *MIRO1*, and *MIRO2* in control or AxD astrocytes. The data were derived from Jones et al., Cell Reports, 2018. **(M,N)** Western blotting detecting CD38, MIRO1, and MIRO2 on WT, R79C, or R239C astrocytes. (Adjusted *p* values in N: *CD38*, WT vs. R79C, 0.2153; WT vs. R239C, 0.0094. *MIRO1*, WT vs. R79C, 0.635; WT vs. R239C, 0.9902. *MIRO2*, WT vs. R79C, 9647; WT vs. R239C,0.9998). Representative results were shown in **M** and the quantification of band intensity was shown in **N**. Data are represented as mean ± SEM. One-way ANOVA followed Dunnett’s multiple comparisons test was applied in **C,** **F,** **G,** **H,** **I,** **J,** **K,** **N**. ^∗∗^*p* < 0.01 and ^∗∗∗^*p* < 0.001.

AxD is a severe leukodystrophy and mainly caused by GFAP mutation (Olabarria and Goldman, 2017). Of all the GFAP mutations identified in AxD patients, R79C and R239C are the most frequently observed mutations (Hagemann et al., 2006). We therefore introduced R79C or R239C mutation into GFAP gene in H1 ESCs using CRISPR/Cas9 and validated the mutations by sanger sequencing (Figure 6A and Supplementary Figure S5J). Then GFAP-mutated, as well as WT, H1 ESCs were differentiated into astrocytes with SOX9 and NFIB as mentioned above. We found that the differentiated astrocytes were positive for S100B and GFAP (Figure 6B), and there was no difference in differentiation efficiency among WT, R79C, or R239C cells (Figure 6C). The notable hallmark of AxD is the presence of Rosenthal fibers, which contain GFAP, alphaB-crystallin (CRYAB), and other components, in astrocytes (Olabarria and Goldman, 2017). Notably, GFAP and CRYAB aggregates were observed in R79C or R239C astrocytes, as well as in R239C heterozygous astrocytes, but rarely detected in WT astrocytes (Figures 6D,E and Supplementary Figures S5K,L), suggesting that astrocytes with GFAP mutations here recapitulated phenocopied the core pathology of AxD.

Then, we examined the mitochondrial properties on WT, R79C, and R239C astrocytes. The mitochondrial copy number calculated as the ratio of mitochondrial DNA (mtDNA) to nuclear DNA (nDNA) (Tarrago et al., 2018) was not altered in astrocytes with GFAP mutation (Supplementary Figure S5A). R79C or R239C mutation in GFAP slightly, but not significantly, increased the MMP as measured by TMRE staining (Supplementary Figure S5B). We did not observe significant difference in mitochondrial morphology or expression of mitochondrial biogenesis and metabolism markers between WT and GFAP-mutated astrocytes, either (Supplementary Figures S5C,D).

Next, we analyzed whether intercellular transfer of astrocytic mitochondria was affected by GFAP mutation. WT or GFAP-mutated astrocytes were divided into two parts and each part was labeled with GFP or mitodsred as above-described (Figure 6F and Supplementary Figure S5M). Then the distinctly labeled astrocytes were co-cultured at a ratio of 1:1. After 24 h, we found that astrocytes with R79C or R239C mutation had a significantly lower transfer efficiency than WT astrocytes (Figure 6F and Supplementary Figure S5E), indicating an impairment of mitochondrial transfer by GFAP mutation. As the lower efficiency might be attributed to impairment in giving or receiving mitochondria, we analyzed mitochondrial transfer from WT or GFAP-mutated astrocytes into SK cells. Astrocytes were labeled with mitodsred and then co-cultured with SK-GFP. After co-cultured for 24 h, mitochondrial transfer into SK from astrocytes with R79C or R239C mutation was less efficient than that from WT astrocytes (Figure 6G and Supplementary Figure S5F). On the contrast, when WT or GFAP-mutated astrocytes were labeled with GFP and then co-cultured with SK-mitodsred, the efficiency of mitochondrial transferred from SK into astrocytes was not significantly different (Figure 6H and Supplementary Figure S5G). Similar results were observed when mouse primary neurons, instead of SK, were used to compare the capacity of WT and GFAP-mutated astrocytes to give (Figure 6I and Supplementary Figure S5H) or receive (Figure 6J and Supplementary Figure S5I) mitochondria. Similarly, R239C heterozygous mutation in GFAP gene also led to a reduced mitochondrial transfer efficiency between astrocytes (Supplementary Figure S5M) and from astrocytes into SK cells (Supplementary Figure S5N), but did not significantly alter the transfer from SK into astrocytes (Supplementary Figure S5O). Taken together, these data indicated that astrocytes with GFAP mutations failed to intercellularly transfer mitochondria as efficient as WT astrocytes, but might normally receive transferred mitochondria.

To explore the mechanism underlying the impaired transfer, we compared the expression levels of *CD38*, *MIRO1*, and *MIRO2* between WT and GFAP-mutated astrocytes. We found that the mRNA expression levels of *CD38* in R79C or R239C astrocytes were significantly lower than that in WT astrocytes, while *MIRO1* or *MIRO2* had a similar expression level between WT and GFAP-mutated astrocytes (Figure 6K). These data were consistent with previously published RNA-sequencing data showing that *CD38* had a lower TPM value in astrocytes differentiated from AxD patient-derived iPSCs, while *MIRO1* and *MIRO2* were not significantly altered (Figure 6L; Jones et al., 2018). Using immunoblotting to detect the protein levels of CD38, MIRO1, and MIRO2, we found that MIRO1 or MIRO2 was not significantly altered by GFAP mutation, while CD38 was significantly lower in R239C astrocytes and slightly, but not significantly, lower in R79C astrocytes (Figures 6M,N and Supplementary Figure S6). Overexpression of CD38 in GFAP-mutated astrocytes failed to rescue the defect in mitochondrial transfer efficiency (Supplementary Figures S5P,Q), which was consistent with the potential necessary, but not sufficient, role of CD38 (Figure 3), and also suggested that some other mediators might be compromised in GFAP-mutated astrocytes.

## Discussion

Previous study demonstrated transfer of mitochondria from astrocytes into neurons (Hayakawa et al., 2016). Here, we further revealed intercellular mitochondrial transfer between astrocytes and from neuronal cells into astrocytes, suggesting that intercellular mitochondrial transfer might prevalently occur. Technically, lentivirus-mediated fluorescent proteins were used here to specifically label mitochondria, avoiding the possibility of cytotoxicity and leakage of fluorescent dyes (Berridge et al., 2018; Berridge et al., 2016). Furthermore, the lentivirus was prepared with the third-generation packaging system, which uses three plasmids consisting of packaging, envelope, and vector constructs separately, thus excluding the possibility of generating replication- or mobilizing-competent virus in the transduced cells (Miyoshi et al., 1998; Tiscornia et al., 2006; Zufferey et al., 1998). Thus, it seemed unlikely that the appearance of dsred-positive mitochondria in GFP-positive cells was because of cross infection of mitodsred lentivirus to the GFP cells during co-culture. Indeed, no GFP signal was detected in the mitodsred-labeled cells, further demonstrating that the cross infection of lentivirus during co-culture might not occur. We found that MMP was elevated in the recipient cells, suggesting that mitochondrial transfer might alter mitochondrial function in the receipt cells. However, transfer of astrocytic mitochondrial into neurons occurs *in vivo* and promotes neuronal survival after stroke (Hayakawa et al., 2016). Whether mitochondrial transfer between astrocytes and from neuronal cells into astrocytes also occurs *in vivo* and more detailed cellular functions of the transfer need further investigation.

CD38 catalyzes the production of cADPR, which mobilizes Ca^2+^ to elicit diverse cellular responses (Wei et al., 2014). Previous studies showed that CD38/cADPR signaling mediated mitochondrial transfer from astrocytes into neurons and from bone marrow stromal cells into multiple myeloma cells (Hayakawa et al., 2016; Marlein et al., 2019). We found that CD38/cADPR signaling also contributed to mitochondrial transfer between astrocytes and from neuronal cells into astrocytes here. MIRO1, one of the two mitochondrial localized Rho GTPases, also regulates mitochondrial transfer from mesenchymal stem cells into airway epithelial cells and cardiomyocytes (Ahmad et al., 2014; Zhang et al., 2016). We found that MIRO1, as well as MIRO2, the other mitochondrial Rho GTPase, also played a role in mitochondrial transfer here, which seems reasonable considering the involvement of the two GTPases in intracellular mitochondrial transport and trafficking (Fransson et al., 2006; Lee and Lu, 2014). Interestingly, both MIRO1 and MIRO2 have two EF-hand Ca^2+^-binding domains (Fransson et al., 2006; Fransson et al., 2003), whether they cross-talked with CD38/cADPR signaling awaits to be explored. Our study, together with previous studies, indicate that there may exist universal mediators for intercellular mitochondrial transfer between different cells. Therefore, it is possible that previously reported mechanisms such as extracellular vesicles, tunneling nanotubes, and gap junctions (Griessinger et al., 2017; Hayakawa et al., 2016; Islam et al., 2012) may also participate in the transfer here.

Using cellular model of AxD, recent studies reveal that GFAP mutations in astrocytes inhibit oligodendrocyte progenitor cell (OPC) proliferation and myelination by secreting CHI3L1 (Li et al., 2018) and disrupt intracellular vesicle regulation and ATP secretion (Jones et al., 2018), taking a step forward to understand how GFAP mutations in astrocytes affect intercellular communication and lead to AxD (Sofroniew, 2018). By introducing R79C or R239C, two hotspot AxD-causative mutations, into the GFAP gene of hPSCs and then inducing astrocyte differentiation, we also generated astrocytes with GFAP mutations as previously described (Canals et al., 2018). We found that intercellular mitochondrial transfer from GFAP-mutated astrocytes was significantly impaired, which might lead to the failure of satisfying the material and energy demands of other neural cells and might also represent the disrupted intercellular communication under pathological conditions. The expression level of CD38 in GFAP-mutated astrocytes tended to be lower than that in WT astrocytes. It is reported that CD38 expression is regulated by TNF-α through NF-κB (Kang et al., 2006; Sun et al., 2006; Tirumurugaan et al., 2008) and TNF-α secretion is increased in AxD astrocytes (Kondo et al., 2016), which appears to contradict our observation. However, even though we are not aware of it, we speculate that except TNF-α and NF-κB, there might also exist some other unrevealed, especially GFAP- related, mechanisms regulating CD38 expression in astrocytes. We found that the restoration of CD38 expression in GFAP-mutated astrocytes failed to rescue the impaired mitochondrial transfer, indicating that some downstream mediators might also be compromised. Besides CD38, the plasticity and dynamics of GFAP filament itself would affect the translocation of functional molecules (such as glutamine synthase, and AQP4) and cellular organelles (such as intracellular vesicles, endoplasmic reticulum, and lysosome) and therefore regulates the activity of adjacent neurons (Jones et al., 2018; Middeldorp and Hol, 2011; Wang and Parpura, 2018). Thus it is also possible that the impaired mitochondrial transfer results from the deficiency in guiding mitochondrial movement of abnormal GFAP filament, during which the CD38/cADPR signaling and MIRO1/MIRO2 might involve and even act as an upstream regulator.

In AxD, the pathological astrocytes lead to the dysfunction of other cell types in central nervous system, including neurons and oligodendrocytes (Olabarria and Goldman, 2017). However, the underlying mechanisms are not fully elucidated. Previous studies indicate that the upregulation of N-cadherin and impairment in ATP release in AxD astrocytes might lead to altered intercellular interaction between astrocytes and other neural cells including neurons and oligodendrocytes (Jones et al., 2018; Kondo et al., 2016). Besides, the decreased ability to buffer glutamate and potassium, as well as elevated levels of cytokines and chemokines, of AxD astrocytes could also result in neuronal dysfunction (such as more sensitive to glutamate-generated death and more depolarized) and oligodendrocyte degeneration (such as toxicity and loss of myelin) (Deng et al., 2006; Olabarria and Goldman, 2017). Meanwhile, AxD-associated GFAP mutations would inhibit the proliferation and terminal differentiation of OPCs (Gomez-Pinedo et al., 2017; Li et al., 2018). Our study here suggested an impairment in intercellular mitochondrial transfer between GFAP-mutated astrocytes and from these astrocytes into neurons. It seems reasonable to speculate that mitochondrial transfer from GFAP-mutated astrocytes into OPCs and mature oligodendrocytes might also be compromised, which needs future investigation. Moreover, it would be interesting to further explore whether the impaired mitochondrial transfer also involves in disrupted proliferation and differentiation of OPCs and demyelination and dysfunction of mature oligodendrocytes. Though we are currently not fully aware of the underlying mechanisms and the functional consequence, the impaired mitochondrial transfer from GFAP-mutated astrocytes might provide a perspective to dissect the pathogenic mechanism in AxD.

## Data Availability

Publicly available datasets were analyzed in this study. This data can be found here: https://www.cell.com/cell-reports/pdf/S2211-1247(18)31543-2.pdf.

## Ethics Statement

The use of animals was approved by the Institutional Animal Care and Use Committee (IACUC) of the Shanghai Institute of Biochemistry and Cell Biology, Chinese Academy of Sciences. C57BL/6 mouse of postnatal day 1 and both genders were used and purchased from the Shanghai SLAC Laboratory Animal Co., Ltd. (Shanghai, China).

## Author Contributions

GP supervised and controlled the research conception and design, interpreted the data, and revised the manuscript. LG organized the figures and drafted the manuscript. JL revised the manuscript. LG, ZZ, and JL conducted the experiments and analyzed the data. All authors contributed to manuscript preparation and approved the submitted manuscript.

## Conflict of Interest Statement

The authors declare that the research was conducted in the absence of any commercial or financial relationships that could be construed as a potential conflict of interest.
