# Supplementary material for: Mitochondria Are Dynamically Transferring Between Human Neural Cells and Alexander Disease-Associated GFAP Mutations Impair the Astrocytic Transfer
Source: Front Cell Neurosci. 2019 Jul 10;13:316. doi: 10.3389/fncel.2019.00316 (PMC6636397; doi:10.3389/fncel.2019.00316)
Supplement: Supplementary file 1 [file Data_Sheet_1.pdf]

Mitochondria are Dynamically Transferring between Human Neural Cells and Alexander Disease-associated GFAP Mutations Impair the Astrocytic Transfer

Longfei Gao[1], Zhen Zhang[1], Jing Lu[1], and Gang Pei[1, 2, 3, *]

[1]State Key Laboratory of Cell Biology, CAS Center for Excellence in Molecular Cell Science, Shanghai Institute of Biochemistry and Cell Biology, Chinese Academy of Sciences; University of Chinese Academy of Sciences, Shanghai, China
[2]Shanghai Key Laboratory of Signaling and Disease Research, Collaborative Innovation Center for Brain Science, School of Life Sciences and Technology, Tongji University, Shanghai, China
[3]Institute for Stem Cell and Regeneration, Chinese Academy of Sciences, Beijing, China

*Correspondence:
Dr. Gang Pei
gpei@sibs.ac.cn

Supplementary Information

Supplementary Figures
Supplementary Figure 1 Intercellular mitochondrial transfer between astrocytes.
Supplementary Figure 2 Mitochondrial transfer from neuronal cells into astrocytes.
Supplementary Figure 3 Contribution of CD38/cADPR signaling and mitochondrial Rho GTPases to the transfer.
Supplementary Figure 4 Effects of mitochondrial transfer on recipient cells.
Supplementary Figure 5 Impaired mitochondrial transfer from astrocytes with GFAP mutations.
Supplementary Figure 6 Uncropped Western Blot images.

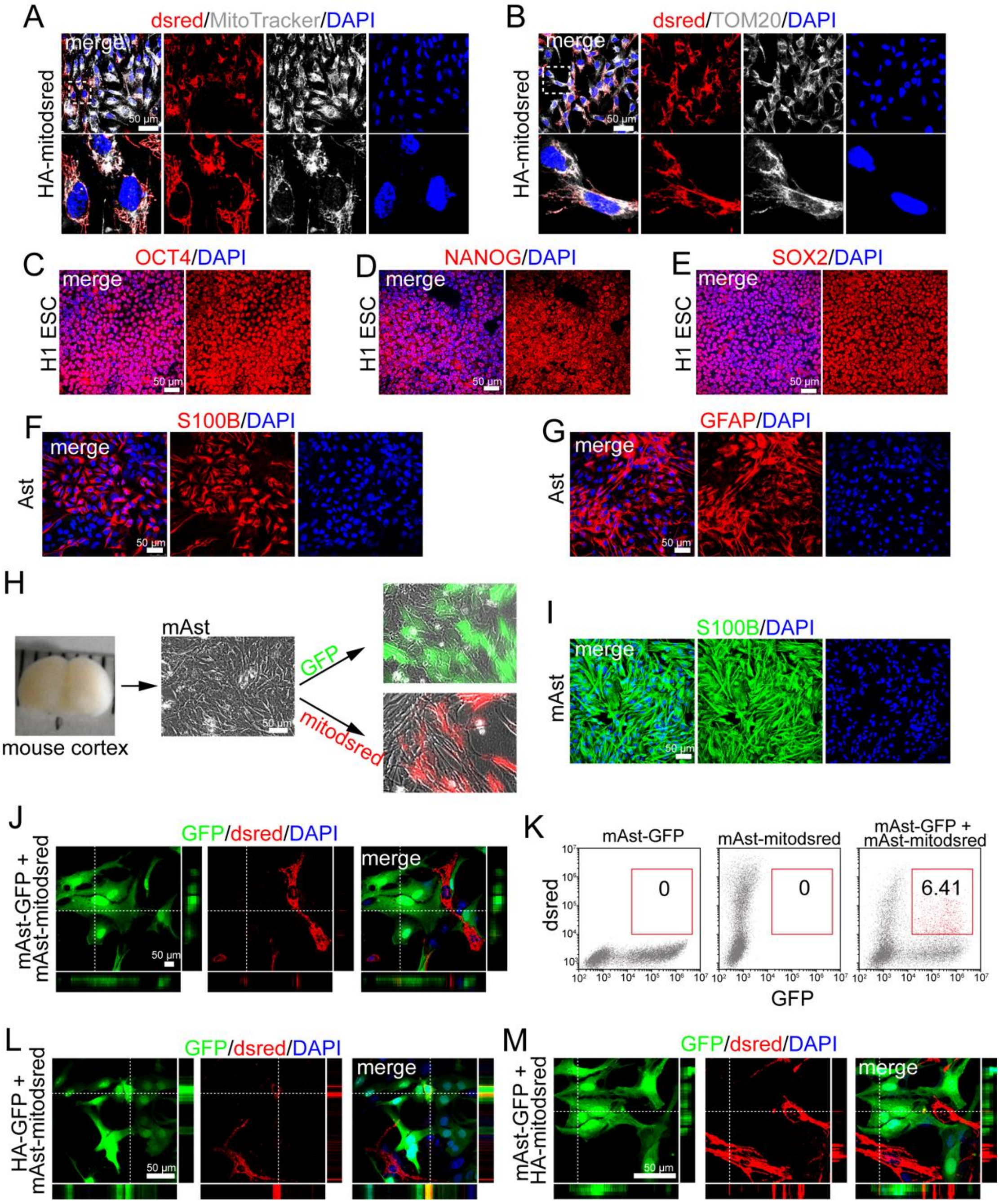

Supplementary Figure 1 Intercellular mitochondrial transfer between astrocytes. **(A-B)** HA-mitodsred were stained with MitoTracker (Deep Red FM) (A) or anti-TOM20 antibody (B). **(C-E)** Immunostaining of OCT4 (C), NANOG (D), and SOX2 (E) on H1 ESCs. **(F-G)** Immunostaining of S100B (F) and GFAP (G) on H1 ESC-derived astrocytes. **(H)** Schematic images showing that primary mouse astrocytes (mAst) were derived from mouse cortex and labeled with GFP or mitodsred lentivirus. **(I)** Immunostaining of S100B on isolated mouse astrocytes. **(J)** mAst-GFP and mAst-mitodsred were co-cultured and mitochondria from mAst-mitodsred were observed in mAst-GFP after co-culture for 24 hours. **(K)** Flow cytometry analyzing mitochondrial transfer efficiency between mAst after co-cultured for 24 hours. **(L)** Mitochondria from mAst-mitodsred were observed in HA-GFP after co-cultured for 24 hours. **(M)**

Mitochondria from HA-mitodsred were observed in mAst-GFP after co-cultured for 24 hours.

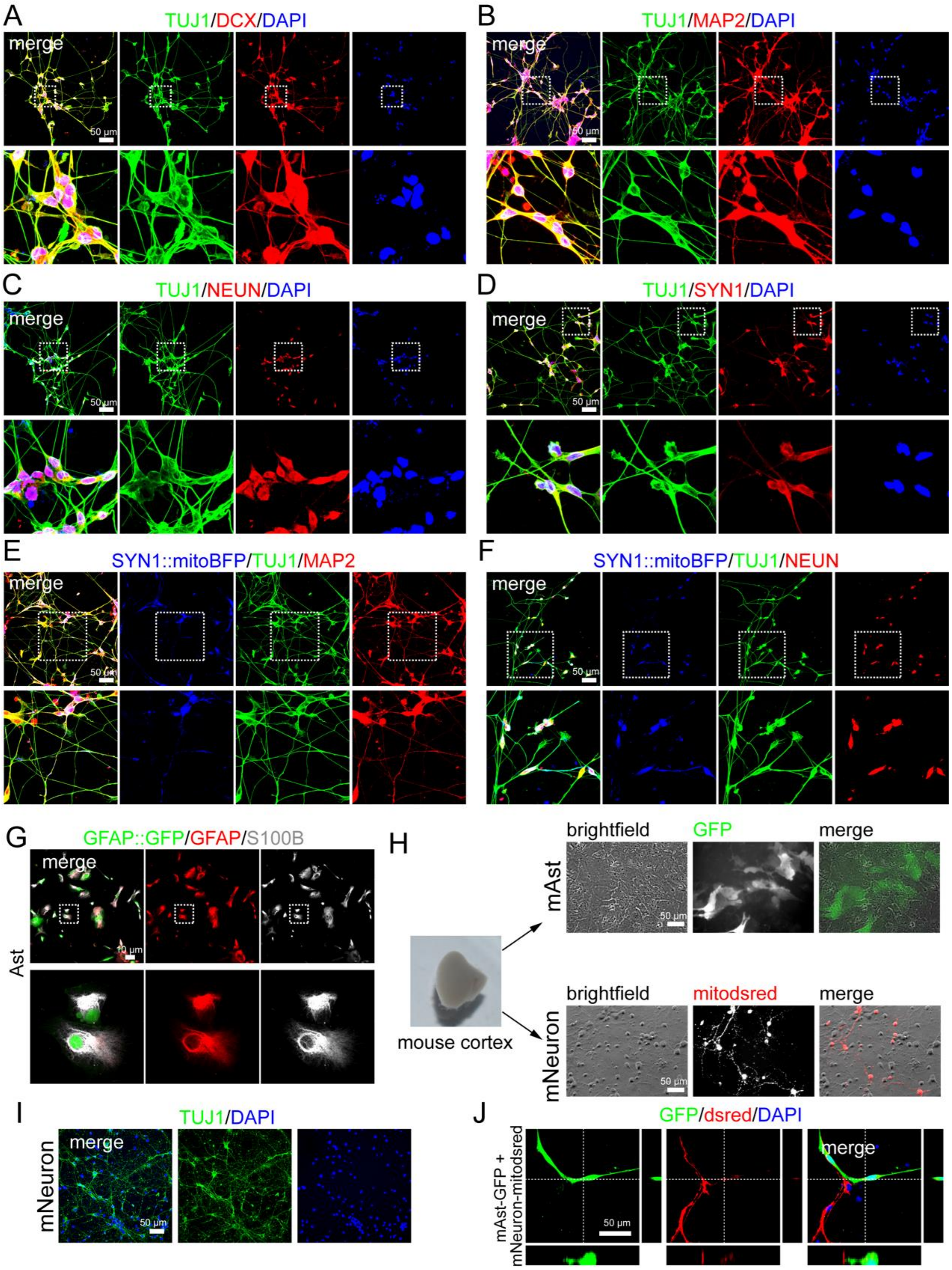

Supplementary Figure 2 Mitochondrial transfer from neuronal cells into astrocytes. (A-D) Immunostaining of TUJ1 (A, B, C, D), DCX (A), MAP2 (B), NEUN (C), and SYN1 (D) on hN. **(E-F)** Immunostaining of TUJ1 (E, F), MAP2 (E), and NEUN (F) on SYN1::mitoBFP labeled hN. **(G)** Immunostaining of S100B and GFAP on GFAP::GFP labeled Ast. **(H)** Primary mouse astrocytes (mAst) or neurons (mNeuron) were derived from mouse cortex. Lentivirus expressing GFP or mitodsred was used to label mAst or mitochondria in mNeuron. **(I)** Immunostaining of TUJ1 on mNeuron. Above 90% cells were positive for TUJ1. **(J)** Confocal images showing that

mitochondria from mNeuron-mitodsred were observed in mAst-GFP after co-cultured for 48 hours.

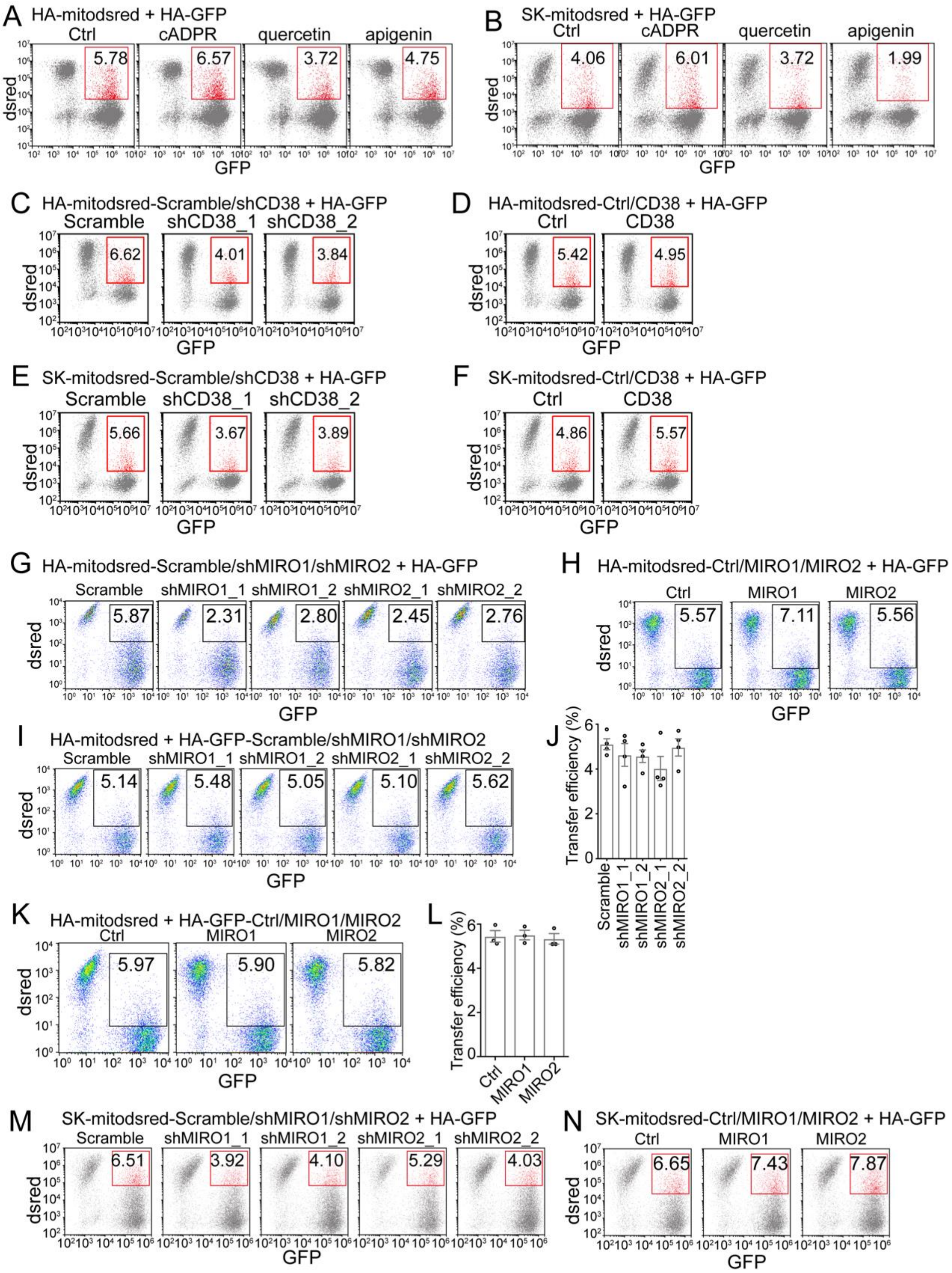

Supplementary Figure 3 Contribution of CD38/cADPR signaling and mitochondrial Rho GTPases to the transfer. (A) Representative flow cytometry results showing mitochondrial transfer between HA with indicated chemical treatment. **(B)** Representative flow cytometry results showing mitochondrial transfer from SK-mitodsred into HA-GFP with indicated chemical treatment. **(C)** Representative flow cytometry results showing mitochondrial transfer from HA-mitodsred transduced with scramble or CD38 shRNA into HA-GFP. **(D)** Representative flow cytometry results showing mitochondrial transfer from HA-mitodsred transduced with CD38 into HA-

GFP. **(E)** Representative flow cytometry results showing mitochondrial transfer from SK-mitodsred transduced with scramble or CD38 shRNA into HA-GFP. **(F)** Representative flow cytometry results showing mitochondrial transfer from SK-mitodsred transduced with CD38 into HA-GFP. **(G)** Representative flow cytometry results showing mitochondrial transfer from HA-mitodsred transduced with scramble, MIRO1, or MIRO2 shRNA into HA-GFP. **(H)** Representative flow cytometry results showing mitochondrial transfer from HA-mitodsred transduced with MIRO1 or MIRO2 into HA-GFP. **(I-J)** Flow cytometry analyzing mitochondrial transfer from HA-mitodsred into HA-GFP transduced with indicated shRNA. (Adjusted *p* values in J: Scramble vs. shMIRO1_1, 0.8182; Scramble vs. shMIRO1_2, 0.7604; Scramble vs. shMIRO2_1, 0.2264; Scramble vs. shMIRO2_2, 0.9971.) **(K-L)** Flow cytometry analyzing mitochondrial transfer from HA-mitodsred into HA-GFP transduced with indicated genes. (Adjusted *p* values in L: Ctrl vs. MIRO1, 0.9776; ctrl vs. MIRO2, 0.9277.) **(M)** Representative flow cytometry results showing mitochondrial transfer from SK-mitodsred transduced with indicated shRNA into HA-GFP. **(N)** Representative flow cytometry results showing mitochondrial transfer from SK-mitodsred transduced with indicated genes into HA-GFP. Data are represented as mean ± SEM. Data are represented as mean ± SEM. One-way ANOVA followed Dunnett's multiple comparisons test was applied in J and L.

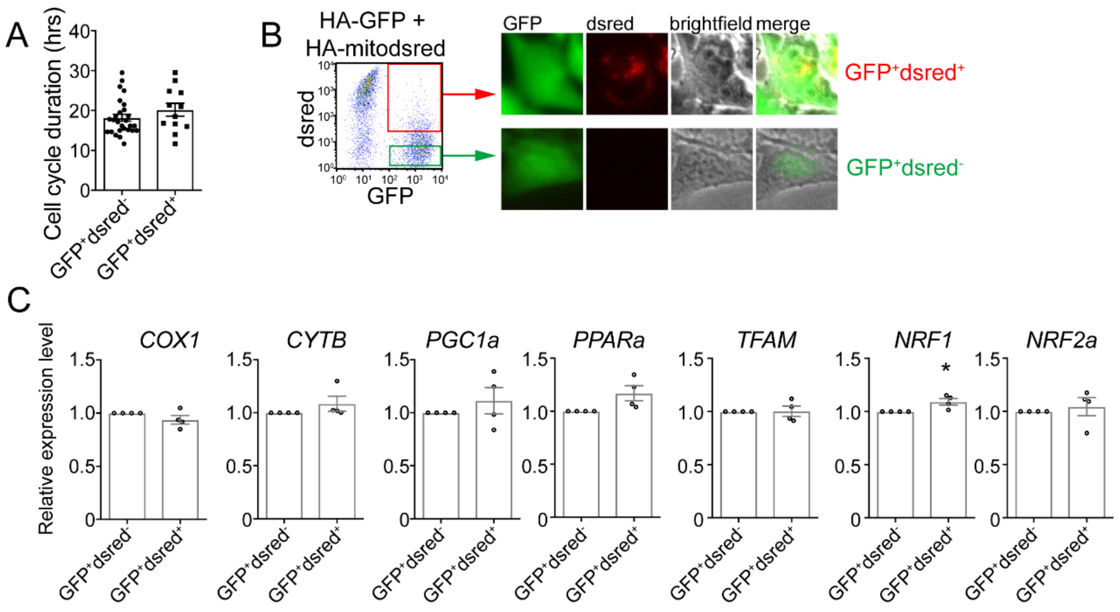

Supplementary Figure 4 Effects of mitochondrial transfer on recipient cells. (A) Comparison of cell cycle duration between HA without transferred mitochondria (GFP$^+$dsred$^-$) and HA with transferred mitochondria (GFP$^+$dsred$^+$). No statistics difference was observed. (p = 0.2427. Two-tailed student t test.) **(B)** Separation of GFP$^+$dsred$^-$ and GFP$^+$dsred$^+$ cells by FACS. **(C)** Comparison of expression levels of mitochondrial biogenesis and metabolism markers between GFP$^+$dsred$^-$ and GFP$^+$dsred$^+$ cells. (p values, *COX1*, 0.1813; *CYTB*, 0.2709; *PGC1a*, 0.3932; *PPARa*, 0.0552; *TFAM*, 0.9474; *NRF1*, 0.0238; *NRF2a*, 0.617. Two-tailed student t test.) Data are represented as mean ± SEM.

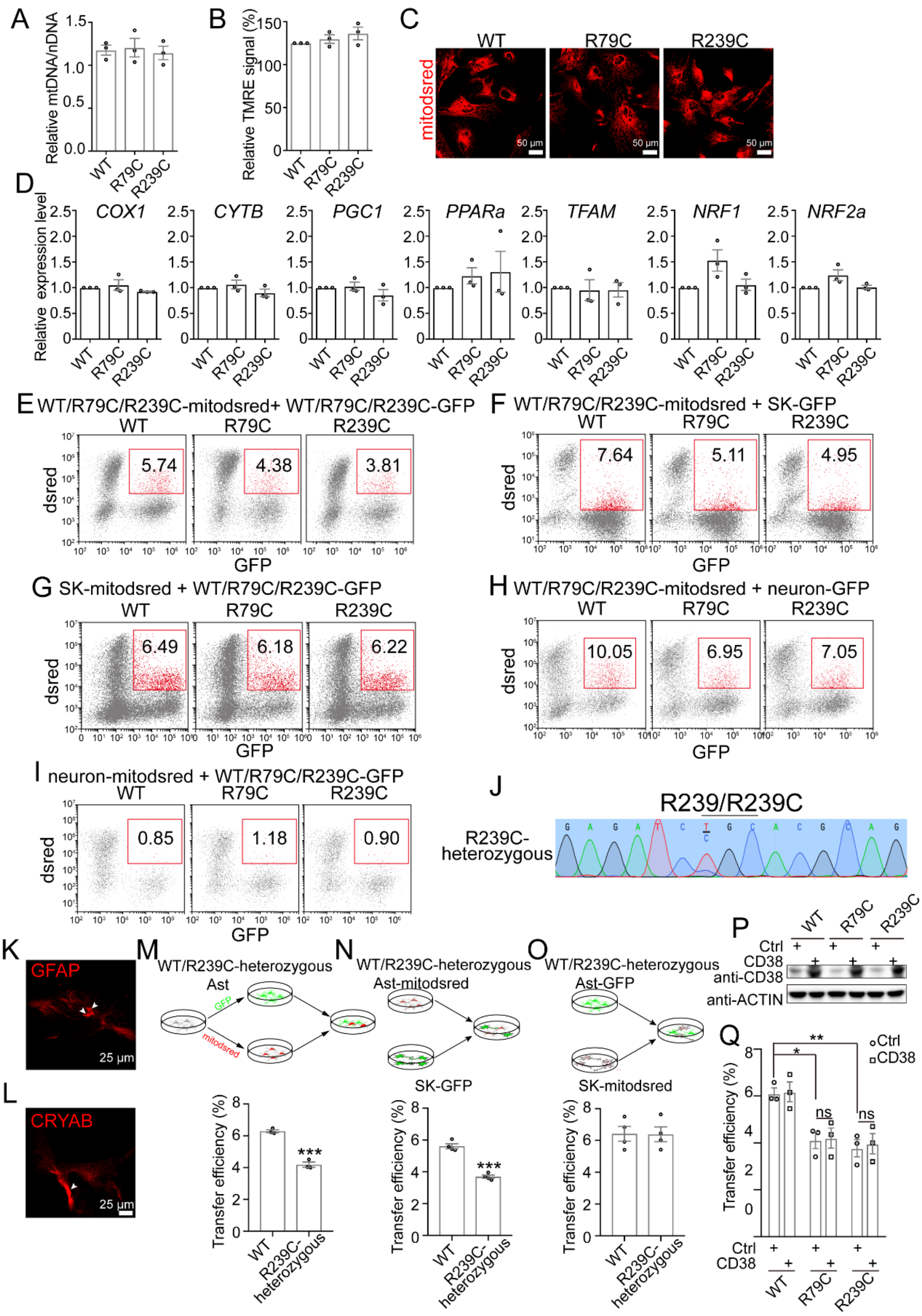

Supplementary Figure 5 Impaired mitochondrial transfer from astrocytes with GFAP mutations. (A-B) Comparison of mitochondrial DNA copy number (A) and MMP (B) among WT, R79C, and R239C astrocytes. (Adjusted *p* values with one-way ANOVA followed Dunnett's multiple comparisons test: A, WT vs. R79C, 0.9576; WT vs. R239C, 0.9477. B, WT vs. R79C, 0.7413; WT vs. R239C, 0.2786.) **(C)** Representative images showing representative mitochondrial morphology in WT,

R79C, or R239C astrocytes. **(D)** Comparison of expression levels of mitochondrial biogenesis and metabolism markers among WT, R79C, and R239C astrocytes. (Adjusted *p* values >0.05, one-way ANOVA followed Dunnett's multiple comparisons test.) **(E)** Representative flow cytometry images showing mitochondrial transfer efficiency between WT, R79C, or R239C astrocytes. Cells co-cultured for 24 hours were analyzed. **(F)** Representative flow cytometry images showing intercellular mitochondrial transfer efficiency from WT, R79C, or R239C astrocytes into SK-GFP. Cells co-cultured for 24 hours were analyzed. **(G)** Representative flow cytometry images showing intercellular mitochondrial transfer efficiency from SK-mitodsred into WT, R79C, or R239C astrocytes. Cells co-cultured for 24 hours were analyzed. **(H)** Representative flow cytometry images showing intercellular mitochondrial transfer efficiency from WT, R79C, or R239C astrocytes into primary mouse neurons. Cells co-cultured for 24 hours were analyzed. **(I)** Representative flow cytometry images showing intercellular mitochondrial transfer efficiency from primary mouse neurons into WT, R79C, or R239C astrocytes. Cells co-cultured for 24 hours were analyzed. (**J**) Sanger sequencing validating R239C heterozygous mutation in GFAP gene in H1 ESCs. (**K-L**) Representative images showing GFAP aggregates (K) and CRYAB (L) (indicated by white arrowheads) in R239C heterozygous astrocytes. **(M)** Flow cytometry analyzing mitochondrial transfer efficiency between WT or R239C heterozygous astrocytes. Cells co-cultured for 24 hours were analyzed (p<0.001, unpaired two-tailed student t test). **(N)** Flow cytometry analyzing intercellular mitochondrial transfer efficiency from WT or R239C heterozygous astrocytes into SK-GFP. Cells co-cultured for 24 hours were analyzed (p<0.001, unpaired two-tailed student t test). **(O)** Flow cytometry analyzing intercellular mitochondrial transfer efficiency from SK-mitodsred into WT or R239C heterozygous astrocytes. Cells co-cultured for 24 hours were analyzed (p=0.9653, unpaired two-tailed student t test). (**P**) Western blotting showing overexpression of CD38 in WT, R79C, or R239C astrocytes. (**Q**) Flow cytometry analyzing mitochondrial transfer between WT, R79C, or R239C astrocytes with or without overexpression of CD38. Cells co-cultured for 24 hours were analyzed (Adjusted *p* values with two-way ANOVA followed Tukey's multiple comparisons test: WT-ctrl vs. R79C-ctrl, 0.0273; WT-ctrl vs. R239C-ctrl, 0.0094; WT-ctrl vs. WT-CD38, 0.999; R79C-ctrl vs. R79C-CD38, >0.999; R239C-ctrl vs. R239C-CD38, 0.999.) Data are represented as mean ± SEM. ns, no significance.

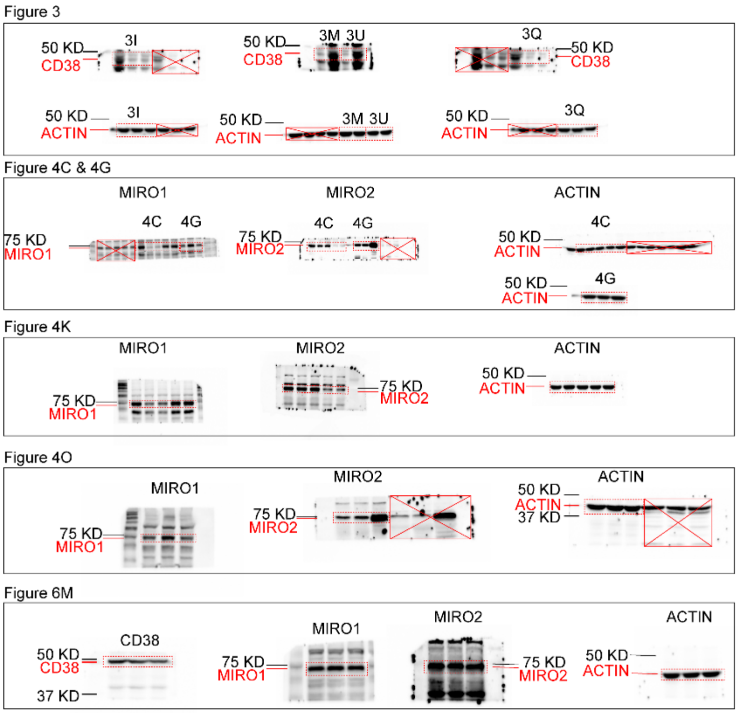

Supplementary Figure 6 Uncropped Western Blot images.
